# Chemokines and Neurodegeneration in the Early Stage of Experimental Ischemic Stroke

**DOI:** 10.1155/2013/727189

**Published:** 2013-11-11

**Authors:** Pawel Wolinski, Andrzej Glabinski

**Affiliations:** ^1^Department of Propedeutics of Neurology, Medical University of Lodz, ul. Pabianicka 62, Lodz, Poland; ^2^Department of Neurology, Epileptology and Stroke, Medical University of Lodz, ul Zeromskiego 113, Lodz, Poland

## Abstract

Neurodegeneration is a hallmark of most of the central nervous system (CNS) disorders including stroke. Recently inflammation has been implicated in pathogenesis of neurodegeneration and neurodegenerative diseases. The aim of this study was analysis of expression of several inflammatory markers and its correlation with development of neurodegeneration during the early stage of experimental stroke. Ischemic stroke model was induced by stereotaxic intracerebral injection of vasoconstricting agent endothelin-1 (ET-1). It was observed that neurodegeneration appears very early in that model and correlates well with migration of inflammatory lymphocytes and macrophages to the brain. Although the expression of several studied chemotactic cytokines (chemokines) was significantly increased at the early phase of ET-1 induced stroke model, no clear correlation of this expression with neurodegeneration was observed. These data may indicate that chemokines do not induce neurodegeneration directly. Upregulated in the ischemic brain chemokines may be a potential target for future therapies reducing inflammatory cell migration to the brain in early stroke. Inhibition of inflammatory cell accumulation in the brain at the early stage of stroke may lead to amelioration of ischemic neurodegeneration.

## 1. Introduction

Stroke is still a major cause of death and long-term disability worldwide and it is associated with significant clinical and socioeconomical problems. Despite the continuous efforts to develop the new pharmacological strategies, there is no effective neuroprotective therapy so far for ischemic stroke. Novel approaches are needed to improve the recovery and quality of life of stroke patients. Development of tissue damage after ischemic insult is dependent not only on duration and intensity of the blood flow reduction, but also on flow independent mechanisms, especially in the peri-infarct brain area. The blood flow dependent mechanisms of tissue damage develop in brain ischemic focus in the short time after onset of blood flow reduction. At that time cell death is a consequence of the acute energy failure and permanent anoxic cells depolarization is induced by loss of ionic gradients. A few hours later, the infarct expands into the adjacent penumbra, and cellular damage is mainly triggered by excitotoxicity, mitochondrial disturbances, reactive oxygen species production, and programmed cell death [1, 2]. 

Excitotoxicity is a pathologic process based on massive activation of AMPA and NMDA receptors in the brain. Inappropriate activation of AMPA and NMDA receptors is a trigger for subsequent dysregulation of calcium ions homeostasis in the neurons and finally results in neuronal loss. Massive activation of those receptors is observed in many CNS disorders including stroke, epilepsy, multiple sclerosis, amyotrophic lateral sclerosis, Parkinson, Alzheimer, and Huntington diseases. The most important factor leading to AMPA and NMDA upregulation is glutamate. Glutamate is an important neurotransmitter in physiological concentration, but in pathologically high concentration it is neurotoxic [3–5]. 

Lately, it is suggested that stroke triggers immune responses leading to inflammatory cell activation and infiltration of cerebral parenchyma. In the stroke brain upregulation of a variety of cytotoxic agents like cytokines, matrix metalloproteinases (MMPs), nitric oxide (NO), and more ROS can be detected [6–8]. There is also upregulation of expression of some chemokines like CCL2 in the CSF [9, 10] and serum of patients with stroke [11]. Studies in experimental stroke (middle cerebral artery occlusion model (MCAo)) confirmed involvement of chemokine CCL2 and its receptors CCR2 in stroke development [12]. Upregulated expression of CCL5 in serum of ischemic stroke patients is controversial. Zaremba et al. reported no difference in the level of CCL5 [13] but Montecucco and colleagues detected increased expression of plasma CCL5 in symptomatic as compared with asymptomatic patients [14]. Moreover, Canouï-Poitrine confirmed that, higher systemic levels of CCL5 and CXCL10 in asymptomatic men are independent predictors of ischemic stroke [15]. There is also a recent report from Tokami et al. supporting the concept that CCL5 may be neuroprotective during stroke development [16]. They showed upregulation of CCL5 but not CCL2, CCL3, and CCL4 on day 0 in stroke patients. This upregulation correlated with plasma concentrations of neuroprotective factors BDNF, EGF, and VEGF [16]. Other data from MCAo model also showed upregulation of several chemokines and their receptors including CCL7 [17], CXCL10 [18], CCL20 [19], and chemokine receptors CXCR4 [20] and CCR6 [19]. 

ET-1 induced model of stroke has been previously described by Anthony et al. who induced the acute rat cerebral blood volume changes after intravenous and intracranial injections of this vasoconstrictor [21, 22]. After microinjection of ET-1 into selected brain regions they observed using magnetic resonance imaging (MRI) an acute reduction of local perfusion in the injected hemisphere, loss of neurons in the grey matter and a macrophage/microglia and astrocyte response. After injection of ET-1 into the cortical white matter, those authors observed amyloid precursor protein-positive immunostaining (indicative of axonal disruption) and an increase in tau-1 immunostaining in oligodendrocytes. Similar to the grey matter lesions, no neutrophils were present and macrophage/microglia response did not occur. Additionally, no breakdown in the blood-brain barrier was detected in the white and grey matter [22]. 

In this study expression of several chemokines including: CCL2, CCL3, CCL5, and CXCL2 as well as expression of markers of neuroinflammation like CD3, F4/80, and IL-1 beta was studied. Correlation of this expression with intensity of early neurodegeneration detected in the brain during the ET-1 induced model of stroke was also analyzed. 

## 2. Material and Methods

### 2.1. Animals

In all experiments, 8- to 12-week-old female SJL/J mice (*n* = five for each time point) were used. All animals were housed at the animal facility of The Medical University of Lodz, Lodz, Poland, under standard conditions. All animals were used in accordance with the Institutional Animal Care and Use Guidelines. All experiments in this study have been approved by the Local Ethics Committee for Affairs Experiments on Animals.

### 2.2. Induction of Endothelin-1 Induced Stroke Model

Animal stroke model was induced by stereotactic, intracerebral injection of endothelin-1 (ET-1, Sigma-Aldrich, Poznan, Poland,) (20 pmol in 1 *μ*L of PBS per mouse) into the left hemisphere of the brain. ET-1 is a potent vasoconstrictor agent of small and large vessels. Prior injection mice were anaesthetized with mixture of ketamine (1,15 mg, Biowet, Pulawy, Poland) and ksylazine (0,1 mg, Biowet, Pulawy, Poland) per mouse. After complete anaesthetization mice were placed in stereotactic frame (David Kopf Instruments, CA, USA), skin on the head was cut, and a small hole in the skull was made using surgical drill. ET-1 was administered with a Hamilton syringe (32G needle) (Hamilton Company, Bonaduz, GR, Switzerland). Site of injection (A-2 mm, L-1, 2 mm, D-2, 5 mm) was selected using the stereotactic atlas “*The Mouse Brain in Stereotaxic Coordinates” Second Edition*, George Paxinos and Keith B.J. Franklin. Tissue samples were collected 24 and 72 hours after the model induction. As a controls, brains from uninjected mice and from mice injected in the same way with PBS were used. 

### 2.3. Extraction of RNA and Proteins

To obtain RNA animals, were perfused with a saline solution. Tissues were weighed and then homogenized using a mechanical homogenizer Ultra Turrax (IKA, Staufen, Germany). Tissues were homogenized in a volume of 1ml of TRIzol LS Reagent (Gibco BRL, Invitrogen, Carlsbad, CA, USA). Homogenates were stored at −80°C until use. RNA was isolated from the homogenates with TRIzol LS Reagent using phenol-chloroform method described by Chomczynski and Sacchi (Chomczynski and Sacchi, 1987). After RNA isolation, its concentration was estimated using the photometric method (BioPhotometr Plus, Eppendorf Company, Wien, Austria). To obtain the proteins for the ELISA assay, the animals were perfused with a saline solution. Harvested organs were weighed using a laboratory balance (Radwag Radom, Poland) and homogenized using a mechanical homogenizer Ultra Turrax (IKA). Homogenisation was performed in a volume of 1 mL HEPES buffer pH 7.4 containing: HEPES −20 mM; EDTA −1.5 mM; benzamidynę −0.5 mM; chicken egg owoinhibitor −10 ug/mL PMSF (phenylmetylsulfonyl fluoride) −0.1 mM (Sigma-Aldrich, Poznan, Poland). After homogenization homogenates were frozen and stored at −80°C. Supernatants were obtained after centrifugation (20 000 × g, time 30 minutes at 4°C MPW, Warsaw, Poland).

### 2.4. Analysis of Gene Expression at the RNA Level by Real-Time PCR

Analysis of the RNA expression was performed using the Corbett Real-Time PCR Machine Rotor Gene 3000 apparatus (Corbett Research, Sydney, Australia). The key enzyme used in this reaction was Taq polymerase with activity of 5 U/mL. Additional reaction components were buffer for polymerase, 25 mm MgCl2, 10 mM dNTPs, fluorescent dye EvaGreen (Biomibo, Warsaw, Poland), 10 *μ*M primers specific to the duplicated sequences, and RNase/DNase free water. For each reaction 2 *μ*L of cDNA derived from the reverse transcription reaction was used and the total volume was 20 *μ*L. As a control histone H3 gene and reference RNA (QPCR Mouse Reference Total cellular RNA, Stratagene, La Jolla, CA, USA) were used.

### 2.5. Analysis of Gene Expression at the Protein Level by ELISA

Quantitative analysis of gene expression at the protein level was performed using ELISA method with commercially available immunoenzymatic Quantikine Kits (R & D Systems, MN, USA). Each set consisted of 96 well plates coated by manufacturer, standard proteins used to prepare the calibration, secondary and tertiary antibodies combined with the horseradish peroxidase enzyme, and washing buffer and color substrate for peroxidase. The assay procedure was performed according to the protocol provided by the manufacturer. After stopping the color reaction protein concentration was evaluated using a photometric reader VICTOR2 Wallac 1420 (PerkinElmer, Waltham, MA, USA) with for 450 nm filters, corrected at 595 nm. All samples were analyzed in duplicates.

### 2.6. Quantitative Assessment of the Level of Neurodegeneration Using ELISA Method

Quantitative assessment of the intensity of neurodegeneration was performed using ELISA method with primary antibodies directed against phosphorylated neurofilaments. The first step was the coating of 96 well Maxisorb Microtitre plate (Nunc, Roskilde, Denmark) with monoclonal anti-NfH antibodies (SMI35R, Sternberger Monoclonals, Convance Princeton, NJ, USA) and overnight incubation at 4°C. Primary antibodies were diluted in carbonate buffer, pH 9.6. The next day tested samples and standard curve samples (neurofilament 200 kD, Progen, Heidelberg, Germany) were added. As a secondary antibody rabbit polyclonal antineurofilament 200 antibody (Sigma-Aldrich, Poznan, Poland) was used. As a tertiary antibody swine antibodies against rabbit immunoglobulin conjugated with horseradish peroxidase (Dako, Glostrup, Denmark) were used. The final step was the addition of color substrate for horseradish peroxidase, which was 3,3′5,5′-tetramethylbenzidine (Sigma-Aldrich, Poznan, Poland). Inhibition of the reaction was performed with 1 M HCl and finally color photometric assessment was done using VICTOR2 reader Wallac 1420 (PerkinElmer, Waltham, MA, USA). The analysis was performed using 450 nm filter with correction at 595 nm. All samples were analyzed in duplicates and the concentration of NfH was determined by referring to the standard curve.

### 2.7. Detection of Localization of Neurodegeneration Using Fluoro-Jade C Dye

Assessment of the localization and severity of neurodegeneration at the level of protein was performed using the fluorescent Fluoro-Jade C dye (Chemicon, Millipore, Warsaw, Poland). Animals were perfused with 4% buffered formalin solution and tissues samples were embedded in paraffin blocks. 10 *μ*m thick sections were applied to a polished Super Frost slides (Menzel-Glaser Braunschweig, Germany). Before final staining paraffin was removed by one hour incubation at 60°C and two 10-minute incubations in xylenes. Tissue was rehydrated in a series of alcohols. Fluoro-Jade C staining was performed according to the protocol provided by the manufacturer (Chemicon). For staining of nuclei sections were counterstained using the blue fluorescent dye DAPI (Sigma-Aldrich, Poznan, Poland). Then the tissue was mounted and coverslipped using DPX (Sigma-Aldrich, Poznan, Poland). 

### 2.8. Image Acquisition

For the analysis and acquisition of images an inverted microscope AxioObserver A1 (Carl Zeiss Inc., Goettingen, Germany) was used. The following lenses made by Carl Zeiss Inc. were used: Plan-Achromat: 4X/0.10, A- Plan 10X/0.25 Ph1; LD A-Plan 20X/0.3; LD Plan-Neofluar 40X/0.6, Ph2 Korr. The images were obtained with a digital camera, AxioCam MRc5 (Carl Zeiss Group, Goettingen, Germany) attached to the microscope. For image acquisition we used Axio-Vision Rel. 4.6 software (Carl Zeiss Group). After obtaining an image no further processing was necessary.

### 2.9. Statistical Analysis

For statistical analysis nonparametric Kruskal-Wallis and Mann-Whitney tests were used. For correlation analyses Kendal tau test was used. A value of *P* < 0.05 was considered statistically significant.

## 3. Results

### 3.1. Expression of Inflammatory Markers in the Brain during the Early Stage of Animal Model of Stroke

**Figure 1:**
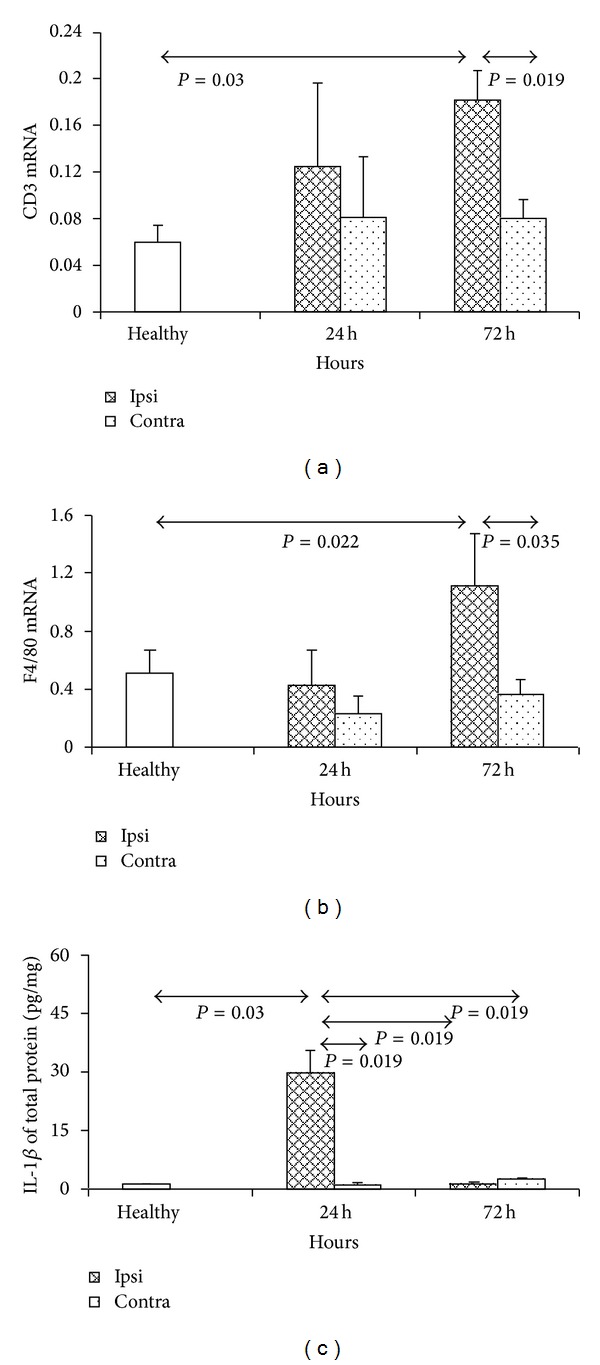
Expression of T cell line marker CD3 (a), macrophage cell line marker F4/80 (b), and inflammatory cytokine IL1 beta (c) in mouse brain during acute stroke model induced by ET-1. The model was induced as described in Materials and Methods. Each analysed group contained 5 mice. Bars represented mean ± SD. Ipsi hemisphere injected with ET-1, contra-contralateral hemisphere, healthy- normal uninjected control.

Significant upregulation of expression of T cell line marker - CD3 was observed in the ET-1-injected hemisphere at 72 h after injection (*P* = 0.03, Mann-Whitney test) (Figure 1(a)). At that time a significant difference in expression of CD3 was detected between ipsilateral and contralateral hemispheres (*P* = 0.019, Mann-Whitney test) (Figure 1(a)). Expression of CD3 in ipsilateral hemisphere was also increased when compared to PBS injected hemisphere (*P* = 0.28, Mann-Whitney test). 

The expression of monocyte/macrophage lineage marker F4/80 was significantly elevated only in ET-1 injected hemisphere at 72 hours after injection. At that time significant difference was observed in expression of F4/80 between ET-1 injected hemisphere and untreated control group (*P* = 0.022; Mann-Whitney test) (Figure 1(b)). We detected also a significant difference in expression of F4/80 between ipsilateral and contralateral hemispheres of ET-1 injected mice at 72 hours after injection (*P* = 0.035, Mann-Whitney test) (Figure 1(b)). 

Upregulation of cytokine IL-1*β* expression was observed in ET-1 injected hemispheres only at 24 h after injection. Significant difference in expression of IL-1*β* was observed between contralateral hemispheres and normal control group at 72 hours after injection (*P* = 0.03 and 0.019, resp.; Mann-Whitney test) (Figure 1(c)). We detected also a significant difference in expression of IL-1*β* between ipsilateral and contralateral hemispheres of ET-1 injected mice 72 hours after injection (*P* = 0.019, *P* = 0.019, resp.; Mann-Whitney test) (Figure 1(c)). 

### 3.2. Expression of Chemokines in ET-1-Induced Stroke Model

**Figure 2:**
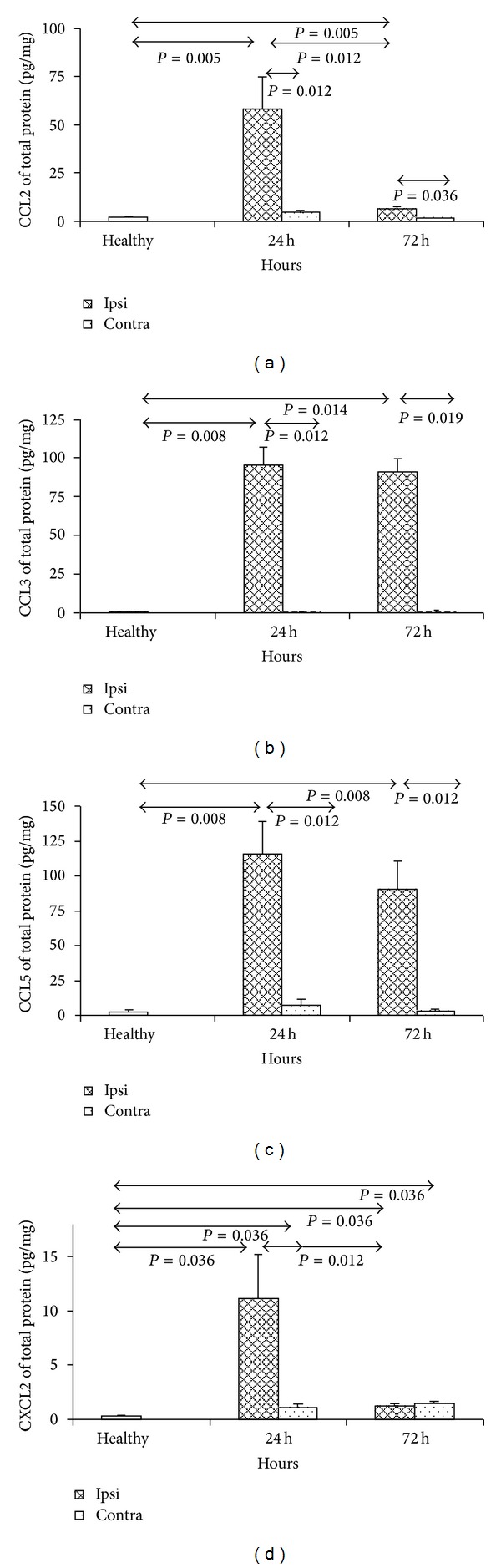
Expression of chemokines CCL2 (a), CCL3 (b), CCL5 (c), and CXCL2 (d) in mouse brain during acute stroke model induced by ET-1. The model was induced as described in Section 2. Each analysed group contained 5 mice. Bars represented mean and ±SD. Ipsi hemisphere injected with ET-1, contra-contralateral hemisphere, healthy- normal uninjected control.

Upregulation of chemokine CCL2 expression was observed in ipsilateral hemispheres at 24 and 72 h after injection of ET-1 (*P* = 0.005, *P* = 0.005, resp.; Mann-Whitney test) (Figure 2(a)). At 24 h CCL2 expression in ipsilateral hemisphere was significantly higher than at 72 h (*P* = 0.019; Mann-Whitney test). Significant difference in CCL2 expression after ET-1 injection was also observed between ipsilateral and contralateral hemispheres at 24 h and 72 h (*P* = 0.012 and 0.036, resp.; Mann-Whitney test) (Figure 2(a)).

We also showed that during early stage of ET-1-injection stroke model expression of CCL3 is significantly upregulated at 24 and 72 h after model induction (Figure 2(b)). There was significant upregulation of CCL3 expression in ipsilateral hemispheres of ET-1 injected mice in comparison to normal controls and contralateral hemispheres at 24 h after injection (*P* = 0.008 and 0.012, resp.; Mann-Whitney test). At 72 h after model induction we observed significant upregulation of CCL3 expression in ET-1-injected hemispheres in comparison to normal brains and contralateral hemispheres (*P* = 0.014 and 0.019, resp.; Mann-Whitney test) (Figure 2(b)). 

Increased expression of the third analyzed inflammatory chemokine—CCL5 was observed in ET-1-injected hemispheres in comparison to uninjected animals at 24 and 72 h (*P* = 0.008, and 0.008 resp.; Mann-Whitney test) (Figure 2(c)). Significant difference was also observed in CCL5 expression between ipsilateral and contralateral hemispheres at 24 h and 72 h after injection of ET-1 (*P* = 0.008 and 0.012, resp.; Mann-Whitney test) (Figure 2(c)). 

Initially increasing expression of CXCL2 in ET-1 injected hemispheres was detected with the peak at 24 h and subsequent decrease at 72 h but still significantly higher than in normal controls (*P* = 0.036, and 0.036, resp.; Mann-Whitney test) (Figure 2(d)). At 24 h CXCL2 expression in ipsilateral hemisphere was significantly higher than at 72 h (*P* = 0.012; Mann-Whitney test) and then at 24 h after ET-1 injection in contralateral hemispheres (*P* = 0.012; Mann-Whitney test) (Figure 2(d)). 

The expression of CXCL12 in the ET-1 and PBS-injected brains and normal controls did not show a significant difference between analysed groups (data not shown).

### 3.3. Analysis of Intensity and Localization of Neurodegeneration in ET-1-Induced Stroke Model

**Figure 3:**
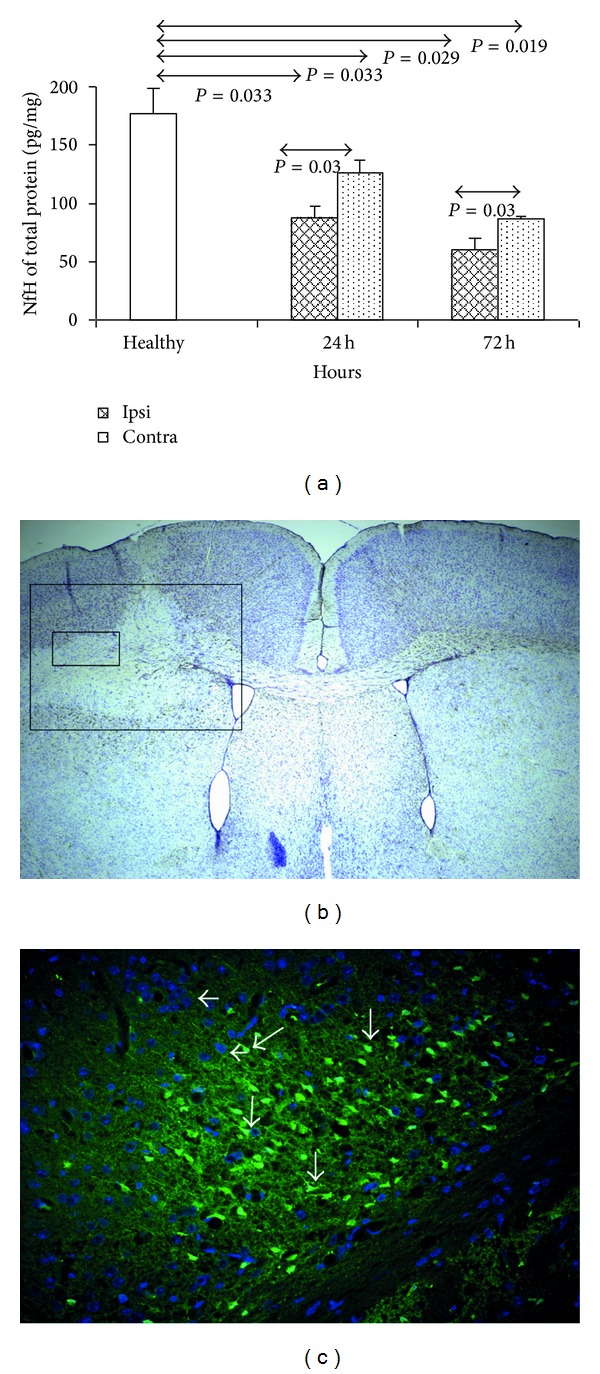
Neurodegeneration in stroke model induced by ET-1. (a) Quantitative analysis of neurodegeneration using ELISA for phosphorylated neurofilaments, (b) localization of neurodegeneration using Cresyl violet staining and GFAP-counterstaining. Large box-stroke area (72 h after ET-1 induction), small box-area showed in C. (c) Localization of neurodegeneration in a stroke model using Fluoro-Jade C staining. White arrows-degenerated neurons. The model was induced and staining performed as described in Materials and Methods. Each analysed group contained 5 mice. Bars represented mean ± SD. Ipsi hemisphere injected with ET-1, contra-contralateral hemisphere, healthy - normal uninjected control.

The most severe neurodegeneration was observed in ET-1-injected hemispheres at 24 and 72 h after model induction (*P* = 0.019 and 0.029, resp.; Mann-Whitney test) (Figure 3(a)). There was also increased neurodegeneration in contralateral hemispheres of ET-1 injected mice at 24 and 72 h, but it was significantly lower than in ipsilateral hemispheres (*P* = 0.03, and 0.03, resp.; Mann-Whitney test) (Figure 3(a)).

The localization of ischemic lesion was detected in ET-1 injected ipsilateral hemispheres using cresyl violet staining (Figure 3(b), large box). Inside the ischemic focus injured neurons were abundant (detected by Fluoro-Jade and marked by arrows) cells nuclei counterstained with DAPI are marked on the picture by arrowheads (Figure 3(c)).

### 3.4. Correlation between Inflammatory Markers and Neurodegeneration in ET-1 Stroke Model

**Figure 4:**
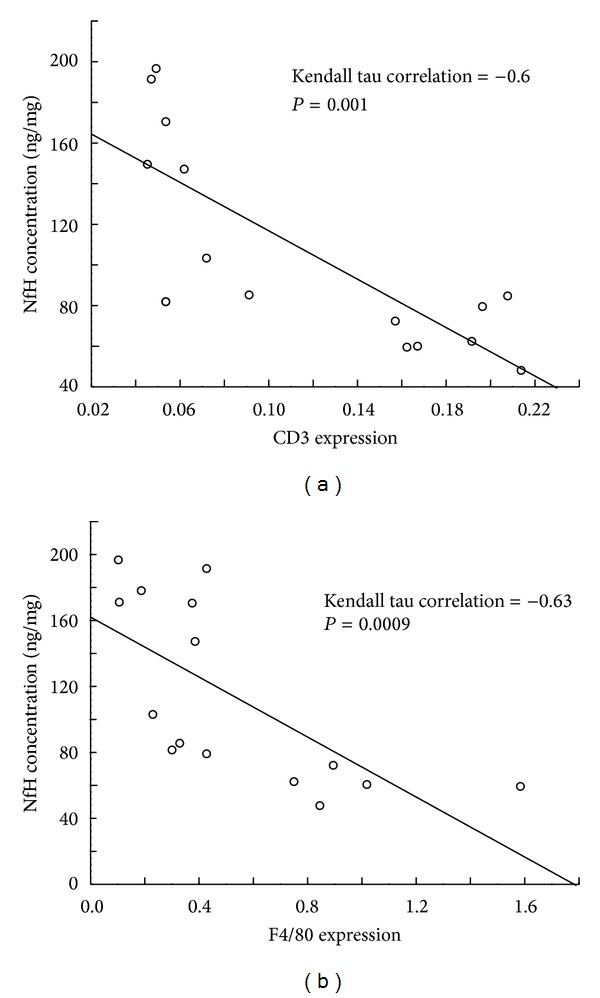
Positive correlation between development of inflammation measured by expression of T cell line marker CD3 (a) and macrophage marker F4/80 (b) with intensity of neurodegeneration measured by ELISA for phosphorylated neurofilaments in ET-1 injected ischemic hemisphere. ET-1 model of stroke was induced as described in Section 2.

We observed the positive correlation between expression of lymphocyte lineage marker CD3 (Kendall Tau = −0,62; *P* = 0.0004) (Figure 4(a)) as well as monocyte/macrophage lineage marker F4/80 (Kendall Tau = −0,56; *P* = 0.0007) (Figure 4(b)) and the severity of neurodegeneration in ET-1 injected brain hemispheres. 

Although the expression of several studied chemotactic inflammatory mediators (chemokines CCL2, CCL3, CCL5, and CXCL2) was significantly increased in the early stage of this stroke model, there was no clear correlation between this expression and intensity of neurodegeneration (data not shown). 

## 4. Discussion

In this study we analyzed potential relationship between neuroinflammation and neurodegeneration in experimental model of ischemic stroke induced by intracerebral ET-1 injection. We focused on a group of proinflammatory chemokines, especially the classical representatives of CCL subfamily. The reason for selecting these chemokines was their confirmed participation in pathogenesis of many central nervous system diseases. During the first few days of the experimental brain ischemia we observed increasing neurodegeneration in ET-1 injected hemisphere. There are several studies showing that neurodegeneration occurs early during brain ischaemia [23, 24]. At that time also inflammatory changes appear in ischemic brain area [25]. The relationship between those two processes is complex and still requires further studies. To measure the intensity of neuroinflammation the expression of inflammatory cells markers (CD3 for T cells and F4/80 for monocytes/macrophages) has been measured at the same time. This analysis showed increased lymphocyte migration to ischemic brain hemisphere at 72 h after model induction. Similarly, infiltration of ipsilateral hemisphere by monocytes/macrophages was significantly increased at 72 h after initiation of brain ischemia. Comparable observation was reported by others who showed the presence of macrophages/activated microglia at 72 h after intracerebral injection of ET-1 to rat brain [22]. In another study using MCAo stroke model, the influx of mononuclear cells to the site of brain ischemia was recorded between 2 and 15 days after model induction [26]. 

The presence of neuroinflammation during early brain ischemia was also confirmed in our study by elevated expression of inflammatory mediator-cytokine IL-1*β*. Its presence was observed as early as 24 h after model induction. Expression of IL-1*β* during brain ischemia has been detected by Barone et al. in several cell types including astrocytes, microglia, neurons, and endothelium [27]. In another study increased production of IL-1*β* was reported even at 3–6 hours after induction of brain ischemia. The peak of this expression was observed at 12 h, and it returned to baseline level after 5 days. Other studies confirmed also that inflammatory mediators, such as IL-1*β* and TNF*α*, are important contributors to CNS neural tissue damage induced by ischemia [28, 29].

In our stroke model analysis of the relationship between the infiltration of ischemic hemisphere by mononuclear inflammatory cells and the intensity of neurodegeneration measured by the presence of phosphorylated neurofilaments showed positive correlation. This may suggest that there is close connection between neuroinflammation and neurodegeneration in ischemic stroke. Migration of inflammatory cells from the blood to the ischemic brain may be at least partially induced by chemotactic cytokines-chemokines. To study this concept we analyzed the expression of some chemokines in the brain. The highest expression of CCL2 was observed in our model at 24 h after initiation of brain ischemia. Increased expression of CCL2 was still observed at 72 h but was at that time significantly lower. Increased expression of CCL2 in MCAo model in the ipsilateral hemisphere was observed on neurons at 12 h and on astrocytes at 24 h after cardiac arrest, suggesting that these cells are the potential source of CCL2 during ischemic stroke [30]. Minami and Satoh using double *in situ* hybridization method pointed to microglia as the cellular source of CCL2 during MCAo [31]. It was shown in another MCAo study that CCL2 leads to infiltration of the CNS by monocytes and thus enhances brain damage induced by ischemia [30]. Also in human stroke patients elevated level of CCL2 was detected in cerebrospinal fluid and serum [9, 11]. 

The highest CCL3 expression was detected at 24 h after ET-1 injection. At 72 h this expression was still increased but it was much lower than at 24 h. Also at the protein level we observed significant increase in CCL3 production in the ischemic hemisphere. Our results are in line with the report by Gourmala et al. who observed an increase in CCL3 expression at mRNA level already at 1 h after MCAo in rats, with peak expression at 8–16 h [32]. In addition, they observed higher expression of CCL3 during temporary MCAo than in permanent MCAo, suggesting the impact of reperfusion on the neuroinflammation in the damaged tissue. Gourmala et al. using *in situ* hybridization localized the expression of CCL3 on microglial cells/macrophages during brain ischemia [32]. In addition, another studies have concluded that CCL3 application to the brain ventricles after complete MCAo enhances MCAo harmful effects [33]. 

We observed almost 46-fold and 30-fold increase in CCL5 expression at 24 h and 72 h, respectively, after induction of ET-1 induced stroke model. There are only a few reports concerning the role of CCL5 in the development of ischemic stroke [15]. Zaremba et al. showed no difference in the level of CCL5 in serum from stroke patients [13]. It was suggested that CCL5 mediates blood-brain-barrier (BBB) disruption and CNS tissue damage as well as inflammation after reperfusion during MCAo model. Terao et al. suggested that platelets are the potential source of CCL5 in rats with MCAo [34]. These data were not confirmed by Tokami and colleagues who observed neuroprotective effect of CCL5 in ischemic stroke suggesting that CCL5 is expressed during stroke mostly in neurons [16]. 

In ET-1 induced experimental stroke a significant increase in CXCL2 expression at 24 h after brain ischemia induction was also observed. This increase returned to the baseline level 48 h later. Rabuffetti et al. observed increased expression of CXCL2 in the brain of rats with the permanent MCAo [35] as well as in the brain and spleen during temporary MCAo in mice. In other study increased CXCL2 expression was observed at 6 h of reperfusion and decreased by almost half at 22 h after reperfusion [36]. Vikman et al. showed increased CXCL2 expression in the brain vessels in the model of subarachnoid haemorrhage and in organotypic cultures [37]. CXCL2 involvement in the inflammatory process in the CNS during MCAo was also confirmed in a SCID mice. MCAo induced in SCID mice led to development of significantly reduced area of brain damage and lower inflammatory infiltration in ipsilateral hemispheres. Reduced expression of many inflammatory mediators including CXCL2 was also observed in T- and B-cell-deficient mice MCAo study [38]. Unfortunately, therapy of ischemic stroke with CXCL2 receptor-CXCR2 antagonists SB225002 was not successful [39]. In report presented by Copin et al. the CXCL1/CXCL2 chemokine-binding protein Evasin-3 treatment was associated with reduction in neutrophilic inflammation in mice MCAo model. However, Evasin-3 administration after cerebral ischemia onset failed to improve poststroke outcomes [40]. 

Although in our study the expression of several studied chemokines was significantly increased at the early phase of ET-1 induced stroke model, no clear correlation of this expression with neurodegeneration was observed. These data indicates that chemokines do not induce neurodegeneration directly. Instead of that they suggest that inhibition of inflammatory cell accumulation in the brain at the early stage of stroke may lead to amelioration of ischemic neurodegeneration. Upregulated in the ischemic brain chemokines may be a potential target for future therapies reducing inflammatory cell migration to the brain in early stroke.

## References

[1] Dirnagl U, Iadecola C, Moskowitz MA (1999). Pathobiology of ischaemic stroke: an integrated view. *Trends in Neurosciences*.

[2] Hossmann K-A (2006). Pathophysiology and therapy of experimental stroke. *Cellular and Molecular Neurobiology*.

[3] Anzai T, Tsuzuki K, Yamada N (2003). Overexpression of Ca^2+^-permeable AMPA receptor promotes delayed cell death of hippocampal CA1 neurons following transient forebrain ischemia. *Neuroscience Research*.

[4] Liu S, Lau L, Wei J (2004). Expression of Ca^2+^-permeable AMPA receptor channels primes cell death in transient forebrain ischemia. *Neuron*.

[5] Yeh T-H, Hwang H-M, Chen J-J, Wu T, Li AH, Wang H-L (2005). Glutamate transporter function of rat hippocampal astrocytes is impaired following the global ischemia. *Neurobiology of Disease*.

[6] Ludewig P, Sedlacik J, Gelderblom M (2013). Carcinoembryonic antigen-related cell adhesion molecule 1 inhibits MMP-9-mediated blood-brain-barrier breakdown in a mouse model for ischemic stroke. *Circulation Research*.

[7] Abdullah A, Ssefer V, Ertugrul U (2013). Evaluation of serum oxidant/antioxidant balance in patients with acute stroke. *Journal of Pakistan Medical Association*.

[8] Hyun H, Lee K, Min KH (2013). Ischemic brain imaging using fluorescent gold nanoprobes sensitive to reactive oxygen species. *Journal of Controlled Release*.

[9] Losy J, Zaremba J (2001). Monocyte chemoattractant protein-1 is increased in the cerebrospinal fluid of patients with ischemic stroke. *Stroke*.

[10] Stowe AM, Wacker BK, Cravens PD (2012). CCL2 upregulation triggers hypoxic preconditioning-induced protection from stroke. *Journal of Neuroinflammation*.

[11] Arakelyan A, Petrkova J, Hermanova Z, Boyajyan A, Lukl J, Petrek M (2005). Serum levels of the MCP-1 chemokine in patients with ischemic stroke and myocardial infarction. *Mediators of Inflammation*.

[12] Sieber MW, Claus RA, Witte OW, Frahm C (2013). Attenuated inflammatory response in triggering receptor expressed on myeloid cells 2 (TREM2) knock-out mice following stroke. *PLoS ONE*.

[13] Zaremba J, Ilkowski J, Losy J (2006). Serial measurements of levels of the chemokines CCL2, CCL3 and CCL5 in serum of patients with acute ischaemic stroke. *Folia Neuropathologica*.

[14] Montecucco F, Lenglet S, Gayet-Ageron A (2010). Systemic and intraplaque mediators of inflammation are increased in patients symptomatic for ischemic stroke. *Stroke*.

[15] Canouï-Poitrine F, Luc G, Mallat Z (2011). Systemic chemokine levels, coronary heart disease, and ischemic stroke events: the PRIME study. *Neurology*.

[16] Tokami H, Ago T, Sugimori H (2013). RANTES has a potential to play a neuroprotective role in an autocrine/paracrine manner after ischemic stroke. *Brain Research*.

[17] Wang X, Li X, Yaish-Ohad S, Sarau HM, Barone FC, Feuerstein GZ (1999). Molecular cloning and expression of the rat monocyte chemotactic protein-3 gene: a possible role in stroke. *Molecular Brain Research*.

[18] Wang X, Li X, Schmidt DB (2000). Identification and molecular characterization of rat CXCR3: receptor expression and interferon-inducible protein-10 binding are increased in focal stroke. *Molecular Pharmacology*.

[19] Terao Y, Ohta H, Oda A, Nakagaito Y, Kiyota Y, Shintani Y (2009). Macrophage inflammatory protein-3alpha plays a key role in the inflammatory cascade in rat focal cerebral ischemia. *Neuroscience Research*.

[20] Schönemeier B, Schulz S, Hoellt V, Stumm R (2008). Enhanced expression of the CXCl12/SDF-1 chemokine receptor CXCR7 after cerebral ischemia in the rat brain. *Journal of Neuroimmunology*.

[21] Sibson NR, Blamire AM, Perry VH, Gauldie J, Styles P, Anthony DC (2002). TNF-*α* reduces cerebral blood volume and disrupts tissue homeostasis via an endothelin- and TNFR2-dependent pathway. *Brain*.

[22] Hughes PM, Anthony DC, Ruddin M (2003). Focal lesions in the rat central nervous system induced by endothelin-1. *Journal of Neuropathology and Experimental Neurology*.

[23] Northington FJ, Ferriero DM, Graham EM, Traystman RJ, Martin LJ (2001). Early neurodegeneration after hypoxia-ischemia in neonatal rat is necrosis while delayed neuronal death is apoptosis. *Neurobiology of Disease*.

[24] Chan KC, Cai KX, Su HX Early detection of neurodegeneration in brain ischemia by manganese-enhanced MRI.

[25] Rodriguez-Grande B, Blackabey V, Gittens B (2013). Loss of substance P and inflammation precede delayed neurodegeneration in the substantia nigra after cerebral ischemia. *Brain, Behavior, and Immunity*.

[26] Kochanek PM, Hallenbeck JM (1992). Polymorphonuclear leukocytes and monocytes/macrophages in the pathogenesis of cerebral ischemia and stroke. *Stroke*.

[27] Barone FC, Arvin B, White RF (1997). Tumor necrosis factor-*α*: a mediator of focal ischemic brain injury. *Stroke*.

[28] Feuerstein GZ, Wang X, Barone FC (1998). The role of cytokines in the neuropathology of stroke and neurotrauma. *NeuroImmunoModulation*.

[29] Chiba T, Itoh T, Tabuchi M (2013). Interleukin-1beta accelerates the onset of stroke in stroke-prone spontaneously hypertensive rats. *Mediators of Inflammation*.

[30] Chen Y, Hallenbeck JM, Ruetzler C (2003). Overexpression of monocyte chemoattractant protein 1 in the brain exacerbates ischemic brain injury and is associated with recruitment of inflammatory cells. *Journal of Cerebral Blood Flow and Metabolism*.

[31] Minami M, Satoh M (2003). Chemokines and their receptors in the brain: pathophysiological roles in ischemic brain injury. *Life Sciences*.

[32] Gourmala NG, Limonta S, Bochelen D, Sauter A, Boddeke HWGM (1999). Localization of macrophage inflammatory protein: macrophage inflammatory protein-1 expression in rat brain after peripheral administration of lipopolysaccharide and focal cerebral ischemia. *Neuroscience*.

[33] Takami S, Minami M, Nagata I, Namura S, Satoh M (2001). Chemokine receptor antagonist peptide, viral MIP-II, protects the brain against focal cerebral ischemia in mice. *Journal of Cerebral Blood Flow and Metabolism*.

[34] Terao S, Yilmaz G, Stokes KY (2008). Blood cell-derived RANTES mediates cerebral microvascular dysfunction, inflammation, and tissue injury after focal ischemiareperfusion. *Stroke*.

[35] Rabuffetti M, Sciorati C, Tarozzo G, Clementi E, Manfredi AA, Beltramo M (2000). Inhibition of caspase-1-like activity by Ac-Tyr-Val-Ala-Asp-chloromethyl ketone induces long-lasting neuroprotection in cerebral ischemia through apoptosis reduction and decrease of proinflammatory cytokines. *Journal of Neuroscience*.

[36] Offner H, Subramanian S, Parker SM, Afentoulis ME, Vandenbark AA, Hurn PD (2006). Experimental stroke induces massive, rapid activation of the peripheral immune system. *Journal of Cerebral Blood Flow and Metabolism*.

[37] Vikman P, Ansar S, Henriksson M, Stenman E, Edvinsson L (2007). Cerebral ischemia induces transcription of inflammatory and extracellular-matrix-related genes in rat cerebral arteries. *Experimental Brain Research*.

[38] Hurn PD, Subramanian S, Parker SM (2007). T- and B-cell-deficient mice with experimental stroke have reduced lesion size and inflammation. *Journal of Cerebral Blood Flow and Metabolism*.

[39] Brait VH, Rivera J, Broughton BRS, Lee S, Drummond GR, Sobey CG (2011). Chemokine-related gene expression in the brain following ischemic stroke: no role for CXCR2 in outcome. *Brain Research*.

[40] Copin JC, da Silva RF, Fraga-Silva RA (2013). Treatment with Evasin-3 reduces atherosclerotic vulnerability for ischemic stroke, but not brain injury in mice. *Journal of Cerebral Blood Flow & Metabolism*.

